# Folic Acid-Grafted Chitosan-Alginate Nanocapsules as Effective Targeted Nanocarriers for Delivery of Turmeric Oil for Breast Cancer Therapy

**DOI:** 10.3390/pharmaceutics15010110

**Published:** 2022-12-28

**Authors:** Htet Htet Moe San, Khent Primo Alcantara, Bryan Paul I. Bulatao, Feuangthit Niyamissara Sorasitthiyanukarn, Nonthaneth Nalinratana, Apichart Suksamrarn, Opa Vajragupta, Pranee Rojsitthisak, Pornchai Rojsitthisak

**Affiliations:** 1 Center of Excellence in Natural Products for Ageing and Chronic Diseases, Chulalongkorn University, Bangkok 10330, Thailand; 2 Pharmaceutical Sciences and Technology Program, Faculty of Pharmaceutical Sciences, Chulalongkorn University, Bangkok 10330, Thailand; 3 Department of Pharmacy, Faculty of Pharmacy, University of Santo Tomas, Manila 1008, Philippines; 4 Department of Food and Pharmaceutical Chemistry, Faculty of Pharmaceutical Sciences, Chulalongkorn University, Bangkok 10330, Thailand; 5 Department of Industrial Pharmacy, College of Pharmacy, University of the Philippines Manila, Manila 1000, Philippines; 6 Metallurgy and Materials Science Research Institute, Chulalongkorn University, Bangkok 10330, Thailand; 7 Department of Pharmacology and Physiology, Faculty of Pharmaceutical Sciences, Chulalongkorn University, Bangkok 10330, Thailand; 8 Department of Chemistry and Center of Excellence for Innovation in Chemistry, Faculty of Science, Ramkhamhaeng University, Bangkok 10240, Thailand; 9 Molecular Probes for Imaging Research Network, Faculty of Pharmaceutical Sciences, Chulalongkorn University, Bangkok 10330, Thailand

**Keywords:** targeted delivery, chitosan-alginate nanocapsules, folate receptor, breast cancer, cytotoxicity, Box-Behnken design

## Abstract

Folate receptors (FRs) highly expressed in breast cancers can be used as a recognized marker for preventing off-target delivery of chemotherapeutics. In this study, folic acid (FA)-grafted chitosan-alginate nanocapsules (CS-Alg-NCs) loaded with turmeric oil (TO) were developed for breast cancer targeting. CS was successfully conjugated with FA via an amide bond with a degree of substitution at 12.86%. The TO-loaded FA-grafted CS-Alg-NCs (TO-FA-CS-Alg-NCs) optimized by Box-Behnken design using response surface methodology had satisfactory characteristics with homogenous particle size (189 nm) and sufficient encapsulation efficiency and loading capacity (35.9% and 1.82%, respectively). In vitro release study of the optimized TO-FA-CS-Alg-NCs showed a sustained TO release following the Korsmeyer-Peppas model with a Fickian diffusion mechanism at pH 5.5 and 7.4. The TO-FA-CS-Alg-NCs showed lower IC_50_ than ungrafted TO-CS-Alg-NCs and unencapsulated TO against MDA-MB-231 and MCF-7 breast cancer cells, suggesting that FA-CS-Alg-NCs can improve anticancer activity of TO through its active targeting to the high FRs expressing breast cancers.

## 1. Introduction

Cancer remains the second leading cause of human mortality and morbidity globally [1,2]. Triple-negative breast cancer (TNBC) is the heterogeneous phenotype of breast cancer, which accounts for 10–20% of all invasive breast cancers and has many molecular subtypes [3]. Current TNBC treatment modalities involve conventional, advanced therapy, and immunotherapy [4]. Drug delivery via uniquely expressed surface biomarkers on TNBC has been identified to augment the cytotoxicity of the therapeutic payload. Among these, the folate ligand has been revolutionized due to its participation in the natural progression of cancer cells and its high affinity toward the folate receptor (FR) [1]. In addition, there is a higher expression of FRs (especially the α-isoform) in breast cancer cells compared to normal cells that can be utilized to develop FR-mediated targeted therapy for breast cancer [1]. 

Recent findings have shown that natural products and their structural analogs have made a promising contribution to pharmacotherapy, especially in treating cancers [5]. Numerous anticancer drugs used in clinics today are either from natural or derived natural products, including plants [6] and microorganisms [7,8]. Among them, Curcuma oil or also known as turmeric oil (TO), derived from the rhizome of *Curcuma longa* from the ginger family, has been proven through the years to have pharmacologic activities including anticancer activity [9]. The major component of TO, *ar*-turmerone was found to have induced apoptosis accompanied by reactive oxygen species (ROS) production [10]. However, the biggest issue of natural products in treating diseases is their pharmacokinetics, specifically their low bioavailability, which hinders clinical success [11]. For instance, patients who have taken orally 3.6 g/day of curcumin will obtain a serum level of 11.1 nmol/L, while those who have received small doses did not have detectable plasma levels.

Nano-based carriers such as lipid-based nanoparticles, polymeric nanoparticles, inorganic nanoparticles, bio-inspired nanoparticles, and hybrid nanoparticles have been used recently to deliver natural products, with the aim of enhancing their pharmacokinetics and pharmacodynamics [11,12]. Nanoparticles can improve the bioavailability and efficacy of natural products in treating diseases due to their small size, large surface area, and increased permeability [13]. Moreover, nanoparticles’ distinctive properties, such as specific targeting of cancerous cells, minimizing the adverse effects, and multi-drug resistance, can be beneficial for cancer therapy [13]. Recently, polymeric nanoparticles have been the primary interest in nano-drug targeting systems. Naturally occurring polymers, such as chitosan (CS) and alginate (Alg), are attractive because of their biological properties, such as non-immunogenicity, biocompatibility, biodegradability, sustained-release property into the bloodstream, or cancerous tissue, and enhanced drug encapsulating efficiency [14,15].

In our previous report, we developed turmeric oil-loaded chitosan-alginate nanocapsules (TO-CS-Alg-NCs) for TNBC [16]. We found that TO-CS-Alg-NCs showed a significant decrease in the half-maximal inhibitory concentration (IC_50_) against MDA-MB-231 and MCF-7 breast cancer cells compared to unencapsulated TO. Ligands that can be attached to the surface of the nanoparticles to increase the cellular uptake and improve the therapeutic efficacy include antibodies, transferrin, aptamers, glycyrrhetinic, sugars (galactose and mannose), folic acid (FA), and peptides (Arg-Gly-Asp or RGD) [17]. The previously developed CS-based nanoparticles for cancer targeting were reported to have particle sizes ranging from 90 to 360 nm [18]. The surface charges of most functionalized CS-based nanoparticles however were found to be near neutral or negatively charged owing to the complexity of the particle system and the addition of new molecules in the CS backbone [18]. Many ligands have been used for CS-functionalization in tumor-targeted delivery including AS1411 aptamer [19], protamine [20], peptides [21,22], and other polymers such as hyaluronic acid [23]. Among these, the folate ligand has been revolutionized due to its participation in the natural progression of cancer cells and its high affinity toward the FR. In addition, there is a higher expression of FRs (especially the α-isoform) in breast cancer cells compared to normal cells that can be utilized to develop FR-mediated targeted therapy for breast cancer [24].

Here, we aim to enhance the anticancer activity of TO-CS-Alg-NCs through the functionalization of the CS with FA for its active targeting strategy to FR-positive TNBC cells. In principle, FA binds to the FR and enters the cancer cells via endocytosis for intracellular drug delivery. Covalently conjugating FA on CS did not interfere with the FR binding and internalization process [1]. Hence, we hypothesized that FA-CS-Alg-NCs could enhance the cytotoxicity of TO against MDA-MB-231 and MCF-7 cancer cells. There is no systematic study that has been reported on the design and optimization of FA-CS-Alg-NCs for encapsulation of TO using statistical design of experiment (DoE). The DoE using response surface methodology (RSM) is a well-trusted statistical approach in investigating and understanding the effects of each variable in the formulation process and achieving the quality that is desirable for a nanocarrier as a drug delivery system [25]. 

In this present study, the FA was grafted on the backbone of CS by the amination-acylation reaction. The resulting FA-CS polymer was characterized by UV-Vis spectrophotometry, 1H-NMR, and FT-IR. The TO-FA-CS-Alg-NCs were fabricated by oil-in-water emulsification followed by ionotropic gelation. The optimization of the TO-FA-CS-Alg-NCs was performed using the RSM with Box-Behnken design (BBD) as the experimental design. The physicochemical properties, morphology, release kinetics, hemocompatibility, and protein binding of optimized TO-FA-CS-Alg-NCs were characterized. The in vitro cytotoxicity of TO-FA-CS-Alg-NCs against MDA-MB-231 and MCF-7 cancer cells was also investigated. The obtained results showed that the TO-FA-CS-Alg-NCs were able to induce significant cytotoxic effects on both cancer cell lines investigated when compared to TO-CS-Alg-NCs without FA. 

## 2. Materials and Methods

### 2.1. Materials

TO containing 12.3% *ar*-turmerone was purchased from Thai-China Flavours and Fragrances Industry (Nonthaburi, Thailand). Standard *ar*-turmerone was provided by the Center of Excellence for Innovation in Chemistry, Faculty of Science, Ramkhamhaeng University (Bangkok, Thailand). Chitosan (CS, MW = 63 kDa, 91.74% DD) was supplied by Marine Bio-Resources, Co., Ltd. (Samut Sakorn, Thailand). Sodium Alg (medium viscosity, A2033) and poloxamer 407 (Pluronic^®^ F-127) were purchased from Sigma-Chemicals (St. Louis, MO, USA). FA and N,N’-dicyclohexyl carbodiimide (DCC) were purchased from Tokyo Chemical Industry, Co., Ltd. (Tokyo, Japan). N-hydroxysuccinimide (NHS) was supplied from AK Scientific Inc. (Union City, CA, USA). Dimethyl sulfoxide (DMSO), acetone, and diethyl ether were purchased from Burdick & Jackson Inc. (Muskegon, MI, USA). Acetonitrile was purchased from RCI Labscan Co., Ltd. (Bangkok, Thailand). Triethanolamine was purchased from Merck KGaA (Darmstadt, Germany). Absolute ethanol, glacial acetic acid, calcium chloride (CaCl_2_), and other chemicals were purchased from Carlo Erba (Val de Reuil, France). 

### 2.2. Cell Culture

Human breast cancer cells, including MDA-MB-231 and MCF-7, were obtained from the American Type Culture Collection (ATCC, Manassas, VA, USA) and were cultured in Dulbecco’s modified Eagle’s medium (DMEM) supplemented with 10% fetal bovine serum and 100 units/mL penicillin/streptomycin in a humidified atmosphere of 5% CO_2_ at 37 °C.

### 2.3. Synthesis of FA-NHS-Ester

The FA ester was synthesized by the following procedure described by Alupei et al. (2017) with modifications [26]. FA (100 mg) was dissolved in 3 mL of DMSO, followed by adding 63 µL of triethylamine as a catalyst. The DCC and NHS were separately dissolved in DMSO and added to the FA solution in 1:2:2 molar ratios of FA:DCC:NHS. The reaction was performed under a nitrogen atmosphere and stirred overnight in the dark due to the photosensitivity of FA. The solution was filtered via a 0.2-µm syringe filter to remove the white precipitate by-product of dicyclohexylurea (DCU). The filtrate was then added dropwise to the cold diethyl ether containing 30% acetone to initiate the precipitation of the yellow FA-NHS-ester product. The precipitate was collected and washed with acetone before drying it in a vacuum desiccator. The final product was stored in a refrigerator for further use.

### 2.4. Conjugation of the FA-NHS-Ester with CS

FA-NHS ester solution in DMSO in a 1:3 molar ratio of FA-NHS:CS was slowly added into the CS solution dissolved in acetate buffer (pH was adjusted to 4.5–4.7 with 1.0 M NaOH) under continuous stirring in the dark at ambient room temperature for 24 h. The reaction was then quenched by adjusting the pH to 9.0 with 1.0 M NaOH before purifying using a dialysis membrane tube with 8–14 kDa molecular weight cut-off against PBS (pH 7.4) for 3 days and deionized water for the next 3 days to remove the unreacted reagents thoroughly. The precipitated yellow product (FA-grafted CS or FA-CS) was lyophilized for 24 h and characterized by UV-Vis spectrophotometry, ^1^H-NMR, and FT-IR [26].

### 2.5. Fabrication of TO-FA-CS-Alg-NCs

TO-FA-CS-Alg-NCs were fabricated by emulsifying TO in poloxamer-Alg aqueous solution followed by ionotropic gelation with FA-CS using the method described by San et al. (2022) [16] with slight modifications. Briefly, an ethanolic TO solution with various concentrations (1–2% *w*/*v*) was added dropwise at the rate of 20 mL/h using a syringe pump into an aqueous Alg solution (20 mL, 0.6 mg/mL, pH 4.9) containing various concentrations of poloxamer (0.5–3% *w*/*v*) under continuous stirring at 1000 rpm. The o/w emulsion was sonicated for 15 min, followed by adding a CaCl_2_ solution (4 mL, 0.67 mg/mL) and an FA-CS solution with various concentrations. After continuous stirring at 1000 rpm for 30 min, the obtained TO-FA-CS-Alg-NC suspension was equilibrated overnight at room temperature before characterization. 

### 2.6. Optimization of TO-FA-CS-Alg-NC Formulation Using BBD

The BBD and RSM were employed to optimize the TO-FA-CS-Alg-NCs. In this study, three factors, including FA-CS:Alg mass ratio (*X*_1_), concentrations of TO (*X*_2_), and poloxamer (*X*_3_), were investigated in various combinations. These factors and their three levels, and the critical attributes of the NCs, namely minimum particle size (*Y*_1_) and maximum values for EE (*Y*_2_) and LC (*Y*_3_), are summarized in (Table 1). The main interaction and quadratic effects were expressed in the polynomial equations. Based on the design, 15 experimental runs were required and randomized to exclude any bias.

### 2.7. Characterization of FA-CS

The FA in FA-CS was quantitatively analyzed using a UV-Vis spectrophotometer (Agilent Carry 60, Agilent Technologies, Santa Clara, CA, USA). Firstly, the calibration curve of standard FA in 0.1 M NaOH was prepared with a concentration range of 2–18 ug/mL (y = 0.0191x + 0.0055; R^2^ = 0.999). Then, 1 mg of FA-CS was dissolved in 10 mL of 2% acetic acid and measured spectrophotometrically at 363 nm [27]. The chemical composition of CS, FA, and FA-CS was characterized by ^1^H-NMR, 400 Hz (Bruker Avance DPX-300, Billerica, MA, USA), and FT-IR (PerkinElmer, Spectrum One, Perkin-Elmer Instruments Co. Ltd., Norwalk, CT, USA). The degree of substitution (DS) of FA to CS was computed using the following equations [27]:

(1) $$\mathrm{DS}\;\left(\%\right)=\frac{\mathrm{Mole}\;\mathrm{of}\;\mathrm{FA}}{\mathrm{Mole}\;\mathrm{of}\;\mathrm{CS}}\times 100\;$$

(2) DS %=cMFAm−c/MCS ×100

where *c* is the amount of FA calculated from the calibration curve, *m* is the amount of FA-CS, *M_FA_* is Mw of FA, and *M_CS_* is the Mw of CS.

### 2.8. Characterization of TO-FA-CS-Alg-NCs

The optimized TO-FA-CS-Alg-NCs were characterized in terms of particle size, polydispersity index (PDI), zeta potential, and morphology. The dynamic light scattering technique was used to measure the size and PDI. The laser doppler electrophoresis technique determined the zeta potential using a Zetasizer (Nano-ZS, Malvern Instruments Ltd., Worcestershire, UK). Transmission electron microscopy (TEM, JEM-2100, JEOL, Tokyo, Japan) evaluated the size distribution and morphology of the TO-FA-CS-Alg-NCs. The EE and LC of the NCs were quantified using ultrahigh-performance liquid chromatography (UHPLC, Agilent 1290 Infinity II LC System, Santa Clara, CA, USA). The TO-FA-CS-Alg-NCs were isolated from the aqueous suspension by ultracentrifugation at 45,000 rpm at 4 °C for 1 h and lyophilized at −85 °C for 24 h. The mass of dried TO-FA-CS-Alg-NCs was recorded, while the amount of TO in the supernatant was quantified using UHPLC. Briefly, the sample was diluted with ethanol and filtered using a 0.45-µm syringe filter before injection to the Intersil^®^ ODS-3 column (4.6 mm × 150 mm, i.d., 5 μm) (GL Sciences Inc., Tokyo, Japan) maintained at 33 °C. The mobile phase was composed of water and acetonitrile (25:75) eluted with an isocratic mode. The injection volume was 20 µL with a 0.5 mL/min flow rate. A diode array detector was used to detect the sample at a wavelength of 254 nm. The total chromatographic run time was about 30 min. The standard *ar*-turmerone was eluted at a retention time of 12.8 min. The quantity of TO in the NCs was computed as the difference between the total amount of TO initially added into the formulation (TO_formulation_) and the amount of turmeric oil present in the supernatant (TO_supernatant_). EE and LC were computed using Equations (3) and (4), respectively:

EE (%) = ((TO_formulation_ − TO_supernatant_)/TO_formulation_) × 100 (3)

LC (%) = ((TO_formulation_ − TO_supernatant_)/Dry mass of NCs) × 100 (4)

### 2.9. In Vitro Release Study

The in vitro release study of TO from TO-FA-CS-Alg-NCs was performed using a dialysis membrane diffusion method based on the procedure reported by Rajkumar, Gunasekaran et al. [28] with some modifications. The dialysis bag was first soaked in the medium for 24 h to remove preservatives before the start of the experiment. Acetate-buffered solution (pH 5.5) and phosphate-buffered saline (PBS) solution (pH 7.4) were prepared according to the British Pharmacopeia 2020 to mimic the tumor and blood environment, respectively. To maintain sink conditions, 40% (*v*/*v*) ethanol was added to the medium. A 20 mL TO-FA-CS-Alg-NC suspension was added to the dialysis bag and sealed with clips on both ends. The dialysis bag was immersed in the releasing medium (500 mL) and maintained at 37 °C under continuous gentle agitation. Samplings were set within 0 to 24 h, wherein 5 mL of medium were withdrawn at specific time points. The withdrawn samples were replaced with an equal volume of fresh medium to maintain sink condition. The concentration of TO in the medium was quantified using UHPLC and calculated against the calibration curve. The cumulative percentage of released TO was calculated using Equation (5):

(5) $$\mathrm{CR}\;\left(\%\right)=\frac{V_{e}\sum_{i=1}^{n-1}C_{n-1}+{\;V}_{o}C_{n}}{m}\times 100$$

where CR is the cumulative amount of TO released (%), V_e_ is the sampling volume (5 mL), V_o_ is the total volume of release medium (500 mL), C_n_ is the concentration of TO at a particular time point (mg/mL), and m is the total amount of TO in TO-FA-CS-Alg-NCs (mg).

The data were fitted to various release kinetic models, i.e., zero-order, first-order, Higuchi, Korsmeyer-Peppas, and Hixson-Crowell, using the DDsolver software, Microsoft Excel plugin program (Version 2010, Microsoft Corporation, Redmond, WA, USA). The best-fit model was chosen based on the highest R^2^ adjusted and model selection criterion (MSC) and the lowest Akaike information criterion (AIC) [29].

### 2.10. In Vitro Protein Binding 

The particle size and zeta potential of nanoparticles can alter after serum protein binding. A modified in vitro protein adsorption method described by Yallapu et al. [30] was used to assess this phenomenon. In brief, 0.1 mg of bovine serum albumin (BSA) powder was dispersed into 1 mL of TO-FA-CS-Alg-NCs suspension and magnetically stirred at 25 °C for 1, 2, 4, 8, and 24 h. The solution was then centrifuged at 10,000 rpm for 15 min to obtain BSA-bound nanoparticles. Finally, the particle size and zeta potential of BSA-bound nanoparticles were measured using the Zetasizer as described above. The measurement was performed in triplicate. 

### 2.11. In Vitro Hemolytic Compatibility

The in vitro hemolytic compatibility of TO-FA-CS-Alg-NCs and free TO was determined in vitro using a hemolysis assay modified by Jiang et al. [31]. In this study, whole blood from a female Sprague Dawley rat provided by the National Laboratory Animal Center of Mahidol University, Thailand, was centrifuged at 3500 rpm for 5 min to obtain red blood cells (RBC), which were then resuspended in PBS (pH 7.4) to prepare RBC suspension (0.1 mL RBC in 0.9 mL PBS). 0.8 mL of TO-FA-CS-Alg-NCs or free TO samples with an equivalent TO concentration of 2.5–10 mg/mL were incubated at 37 °C with 0.2 mL of RBC suspension in an Eppendorf tube. After 15 min of incubation at 37 °C, the samples were centrifuged at 3500 rpm for 5 min, and free hemoglobin in the supernatant was measured for optical density at 577 nm using a microplate reader (CLARIOstar^®^, BMG LABTECH, Ortenberg, Germany). PBS (pH 7.4) and ultrapure water were used as the negative (non-hemolysis) and positive (hemolysis) control groups, respectively. The hemolysis percentage was calculated using the following Equation (6)

Hemolysis (%) = [(A_sample_ − A_negative_)/(A_positive_ − A_negative_)] × 100% (6)

where A_sample_, A_negative_, and A_positive_ represented the absorbance of the sample, negative control, and positive control group, respectively.

### 2.12. Determination of Cell Surface FR Expression

To determine the expression of FRs on the cell surface, each cell line was seeded at a density of 10 × 10^4^ cells per 500 µL into each well of 24-well flat-bottom culture plates and incubated for 24 h using the protocol stated in Section 2.2. The cells were washed with PBS, detached from the plate by adding 2.9 mM EDTA in PBS, and incubated at room temperature for 8 min. The EDTA solution was removed by centrifugation of cell suspension at 1800 rpm for 5 min at 4 °C. Cells were washed twice with PBS and blocked with 0.5% BSA in PBS. Cells were stained with R-phycoerythrin-conjugated anti-human FRs by incubating at 4 °C in the dark for 30 min. Mouse IgG control antibody was used as an isotype control. Cells were washed twice with PBS. Stained cells were acquired on a BD Accuri C6 (BD Biosciences, Franklin Lakes, NJ, USA) flow cytometer, and data were analyzed using FlowJo™ v10.8 Software (BD Life Sciences, USA).

### 2.13. Cellular Uptake Study

Since TO has no fluorophore, the fluorescein-5-isothiocyanate (FITC, Energy Chemical Shanghai, China) was used as the fluorescent probe by encapsulating it into the FA-CS-Alg-NCs for qualitative cellular uptake detection. The FITC-FA-CS-Alg-NCs were prepared using the optimal conditions in preparing TO-FA-CS-Alg-NCs. MDA-MB-231 cells (high FR expression cells) and normal breast cancer MCF-10A cells (low FR expression) were cultured at a density of 2 × 10^4^ cells/well in a 4-well chamber slide and further incubated for 24 h. Cells were treated with FITC-FA-CS-Alg-NCs in serum-free media or blank FA-CS-Alg-NCs for 15 min and 1 h. To confirm the importance of FR expression, cells were also pre-treated with 1.5 µg/mL anti-human FRαβ antibody (Biolegend) for 30 min prior to FITC-FA-CS-Alg-NCs treatment. Cells were washed 3 times with phosphate buffer saline and fixed by adding 4% paraformaldehyde for 20 min. Cells were stained with DAPI for nuclear staining. The uptake of FITC-FA-CS-Alg-NCs was determined using an inverted fluorescence microscope (Olympus IX51, Olympus Corp., Tokyo, Japan).

### 2.14. Cytotoxicity Assay

To determine the cytotoxicity of TO-FA-CS-Alg-NCs against MDA-MB-231 and MCF-7 cell lines, the cells were seeded at a density of 3 × 10^4^ cells per 100 µL into each well of 96-well culture plates and incubated at 37 °C for 24 h. The cells were then treated with 5 serial concentrations of pure TO and TO-FA-CS-Alg-NCs in a serum-free media and incubated at 37 °C for 24 h. The blank FA-CS-Alg-NCs (without TO) served as the control. After 24 h of treatment, the culture media was removed, and 100 µL of an MTT reagent (0.5 mg/mL in serum-free media) was added to each well and further incubated at 37 °C for 4 h. The MTT media was removed, and the insoluble formazan crystals were dissolved by adding DMSO. After completely dissolved, the absorbance was measured at 570 nm using a microplate reader (CLARIOstar, BMG Labtech, Ortenau, Baden-Württemberg, Germany). The cell viability was calculated using Equation (7):

Cell viability (%) = (OD_sample_/OD_control_) × 100 (7)

### 2.15. Storage Stability Study

The storage stability of TO-FA-CS-Alg-NCs was evaluated by assessing particle size and EE. The optimal TO-FA-CS-Alg-NC nanosuspensions were kept at different storage temperatures (4 °C and 25 °C) for 3 months.

### 2.16. Statistical Analysis

Three independent replicates were utilized in all experiments, and the collected data were expressed as mean ± standard deviation. The polynomial equations were generated using multiple linear regression followed by model-fitting with one-way ANOVA using the Design-Expert^®^ software 13.0.11.0 (Stat-Ease, Inc., Minneapolis, MN, USA). The cell viability results were analyzed using two-way ANOVA with Tukey HSD as the post hoc test. The IC_50_ values for the unencapsulated TO and TO-FA-CS-Alg-NCs were determined through a non-linear regression curve fit analysis. All statistical parameters of the cellular assays were generated using GraphPad^®^ Prism software 9.3.0 (San Diego, CA, USA). The *p*-values < 0.05 were considered statistically significant. 

## 3. Results and Discussion

### 3.1. Synthesis and Characterization of FA-CS

**Figure 1:**
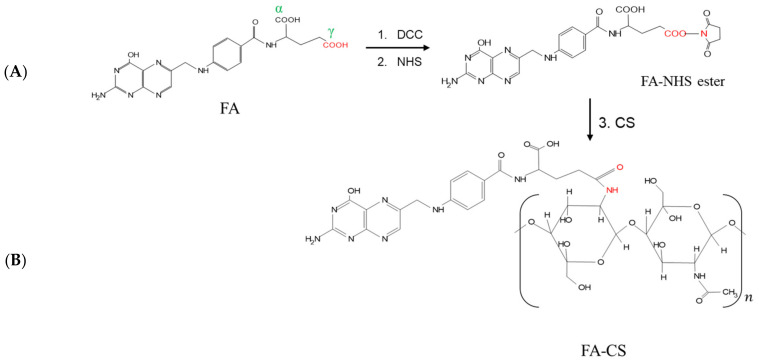
Reaction scheme for the synthesis of (**A**) FA-NHS-ester using DCC and NHS in DMSO, 24 h, room temperature; (**B**) FA-CS using the FA-NHS ester and CS solution, 24 h, room temperature.

The conjugation of FA and CS involved the DCC/NHS reaction (Figure 1). Even though FA has two carboxyl groups (α and γ), γ-COOH of FA is more prone to this reaction due to its stronger electrophilic and less steric properties [32]. The carboxylic group was activated with DCC and NHS to form the FA-NHS ester and its by-product DCU. The final product, FA-CS conjugate, was obtained by forming an amide bond between the activated carbon of FA-NHS ester and the primary amino group of CS. The DS of the FA-CS was 12.86%.

**Figure 2:**
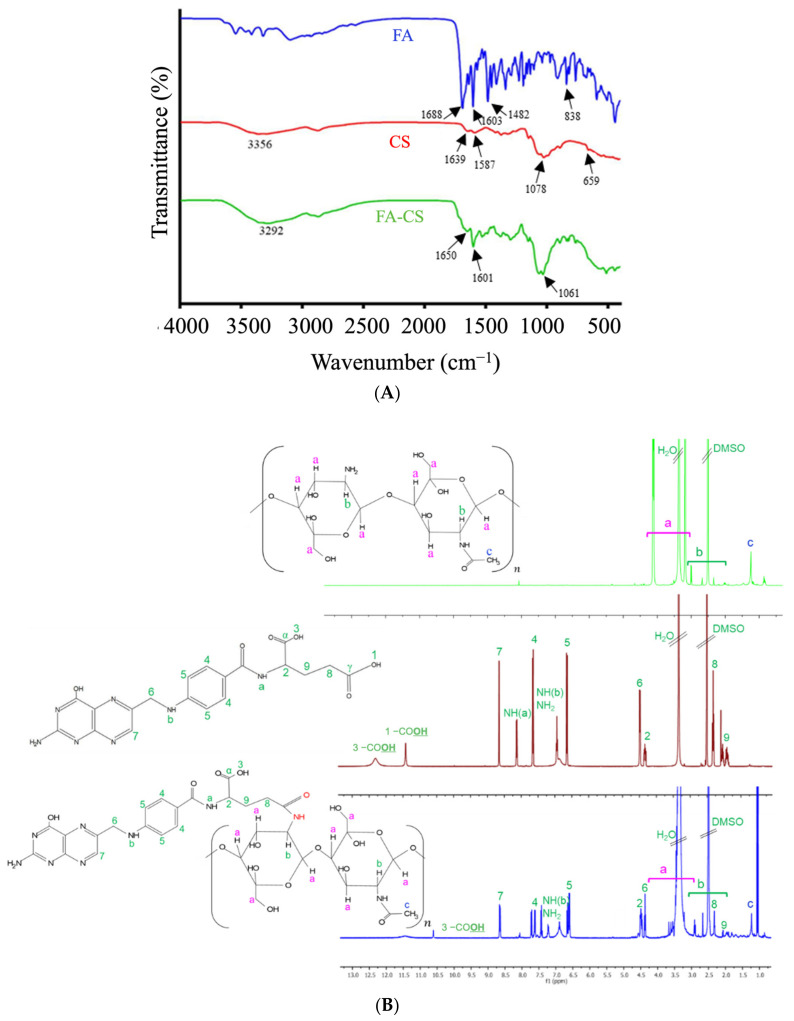
Characteristics of FA, CS, and FA-CS conjugates: (**A**) FT-IR spectra; (**B**) ^1^H-NMR spectra.

The conjugation between CS and FA to yield FA-CS was confirmed by FT-IR (Figure 2A). The spectra in FA exhibited the bond vibration of C=O and the C=C (aromatic) stretching at 1688 cm^−1^ and 1603 cm^−1^, respectively. The band at 1482 cm^−1^ represented the stretching C=C, and at 838 cm^−1^ is a characteristic band of the para-substituted benzene ring [27,33]. Moreover, the CS spectrum ascribed the O-H and N-H stretching vibration of –OH and –NH_2_ functional groups at 3356 cm^−1^ while at 1639 cm^−1^ and 1587 cm^−1^ represented C=O stretching and N-H bending mode of the amide (N-acetylated CS) and 1° amine, C-O stretching at 1078 cm^−1^ and peak at 659 cm^−1^ for the reflecting the pyranoside ring stretching vibration [34]. After FA was conjugated with CS, priority bands related to the functional groups of FA and CS suggested the successful conjugation between FA and CS. Comparing the CS spectrum, the bands at 1587 cm^−1^ and 1639 cm^−1^ shift to 1601 cm^−1^ and 1650 cm^−1^, which were assigned to aromatic C=C from the conjugated FA, –C=O of the amide bond and N-H bending of the 2° amine in each in FA-CS spectra. The 1650 cm^−1^ peak had a higher transmission than the 1601 cm^−1^ peak in the FA-CS. These results correspond to a less amide formation, consistent with the percentage grafting of FA-CS at 12.86%. The FT-IR results indicate that the –COOH group of folic acid was successfully conjugated with the –NH_2_ group of CS. Furthermore, a band at 3292 cm^−1^ was broader due to an enhanced hydrogen bonding between FA and CS [33,34].

The conjugation of FA and CS was structurally confirmed using ^1^H-NMR (Figure 2B and Table S1). The signals at 2.50 and 3.30 ppm belong to the DMSO-*d*_6_ and water, respectively. Based on the FA spectrum, the characteristic peak at 11.40 ppm was attributed to the –OH proton in the γ-carboxylic group, whereas the peak at 12.30 ppm corresponded to the hydroxy proton of the pteridine ring of FA. The signal at 8.13 and 6.63 ppm were assigned to the –NH proton of glutamic acid and pteroic acid of FA, each in order. The peak at 1.24 ppm of the CS spectrum corresponded to the hydrogen of the methyl group of N-acetyl glucosamine. However, in the spectrum of FA-CS spectrum, the signal between 2.60–3.80 ppm belongs to the carbon protons of the glucosamine ring of the CS. Some of the signals were overlaid by the influence of the solvents and the interactions between the two reactants. Finally, the FA was successfully conjugated with CS by inspecting the signal of the FA spectrum at 11.40 ppm that disappeared, and 12.30 ppm was shifted to 10.60 ppm in the FA-CS spectrum.

### 3.2. Assay of ar-Turmerone Content

The main constituents of TO are bisabolene sesquiterpenes consisting of *ar*-turmerone, curlone, α-turmerone, β-turmerone and bisacumol [35]. In this study, the calibration curve of standard *ar*-turmerone used was performed at the wavelength of 254 nm with a linear equation (y = 86.082x + 10.617, R^2^ = 0.9999). Furthermore, commercial turmeric oil was quantified by using this equation. Commercial turmeric oil contains 12.3% of *ar*-turmerone. The EE of the nanoformulation was also computed based on that equation.

### 3.3. Model Development for Particle Size, EE, and LC

The optimization of the formulation process was done by employing the statistical design of the experiment. In this study, a total of 15 experimental runs were constructed from a 3-factor, 3-level BBD of the experiment with triplicated center points. A triplicated center point is optimal to provide excellent prediction capability. The 3 factors (FA-CS:Alg mass ratio, TO, and poloxamer concentrations) were chosen because they primarily affected particle size, EE, and LC based on the literature and preliminary experiments. Various ratios of the FA-CS:Alg were investigated during the preliminary experiments to determine the maximum working formulation ratio. It was found that the mass ratio of 0.07:1 resulted in a dispersion that appeared translucent and no precipitation after 24 h of standing, indicating its stability. Increasing the amount of FA-CS resulted in a visible gel in the suspension. Previously, it was demonstrated that increasing the quantities of CS or Alg increased the particle size and the PDI of the NPs and decreased the stability due to the increased viscosity of the dispersion and the presence of an excess amount of polymer by forming clusters of particles [36]. Increasing the polymer ratio could render a more permeable and porous coating, resulting in a reduced entrapment of the hydrophobic compound and subsequent lowering of EE. Decorating the polymeric NP’s surface with folate and encapsulating a hydrophobic compound has also shown a higher EE than the plain polymeric NPs [32]. On the other hand, increasing the concentration of hydrophobic compounds like TO could gradually increase the particle size and LC [37]. Finding the optimum condition for the TO concentration was, therefore, important. Based on the preliminary experiments, the TO concentration greater than 2% resulted in a turbid dispersion.

The concentration of poloxamer was also considered in the design of the experiment. The preliminary investigations have shown that the maximum working concentration of poloxamer was 3%. Bhunchu et al. (2015) also demonstrated that an increase in poloxamer concentration led to an increase in the entrapment of hydrophobic compounds; however, a further increase in poloxamer concentration resulted in a decrease in EE and LC [38]. This might be due to the poloxamer increasing the viscosity of the system and partitioning the drug into the aqueous phase [29].

Particle size was a critical response in determining the applicability of the optimized NCs for IV administration. Nanoformulations with sizes below 200 nm would have an efficient passive tumor targeting through the enhanced permeation and retention effect and serve as the first of a series of steps in the cellular uptake of the NPs [39]. To ensure a successful biological activity, the other two responses, EE and LC, which represented the level of TO in the nanoformulation, should also be statistically optimized using BBD-RSM.

The advantage of using BBD-RSM is its ability to perform multifactorial analysis on each response with fewer experimental conditions [40]. The experimental ranges of the factors were determined through literature reviews and preliminary experiments. The observed responses for the optimization of the TO-FA-CS-Alg-NCs are shown in Table 2. The particle size, EE, and LC of the TO-FA-CS-Alg-NCs ranged from 175 to 365 nm, 16.9 to 38.3%, and 0.69 to 5.53%, respectively.

The polynomial equations were generated using multiple linear regression to determine the relationship between the factors and the responses. Various statistical parameters were utilized to determine the equations that fit the data well, such as the *p*-value of the overall model, lack-of-fit, R^2^, adjusted R^2^ and predicted R^2^, and adequate precision based on the ANOVA. Based on the *p*-value of each regression coefficient, the model reduction was performed to refine and increase the precision of the models by retaining only the significant terms (*p*-value < 0.05). The results of the regression analyses are presented in Table S2. The overall models show that the coefficients were statistically significant jointly (*p* < 0.0001), indicating that the factors in each model improved its fit. The lack-of-fit test for each model suggests an insignificant lack-of-fit of the regression model (*p* > 0.05), showing a linear relationship between the factor and response. There was also a reasonable agreement between the adjusted R^2^ and predicted R^2^, having a difference of less than 2. The adequate precision of the models, measuring the signal-to-noise ratio, were all greater than 4, indicating that the design space can be navigated with precision.

**Figure 3:**
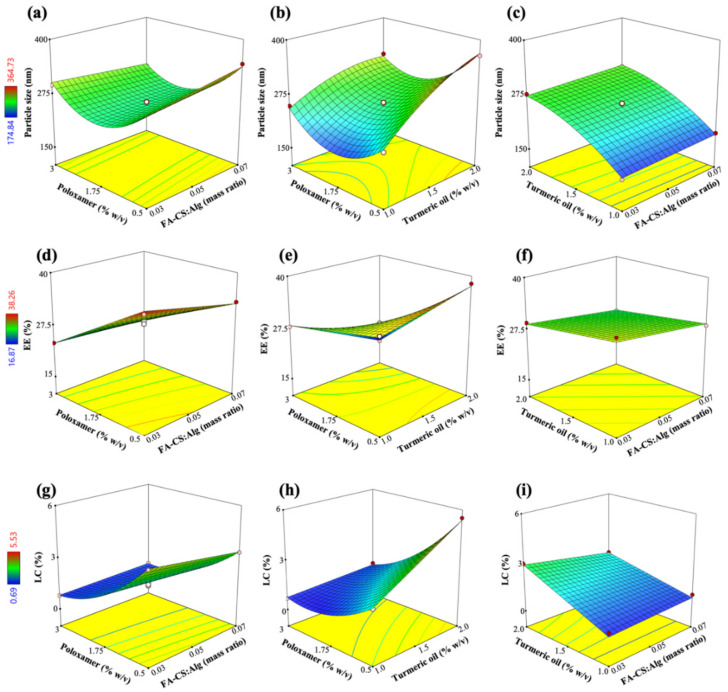
Response surface plots showing the effects of FA-CS:Alg mass ratio (*X*_1_), turmeric oil concentration (*X*_2_), and poloxamer concentration (*X*_3_) on (**a**–**c**) particle size (*Y*_1_), (**d**–**f**) EE (*Y*_2_), and (**g**–**i**) LC (*Y*_3_).

The well-fitted particle size, EE, and LC models were quadratic, 2-factor interaction (2FI), and quadratic, respectively (Equations (8)–(10)). The 3D surface plots for *Y*_1_ to *Y*_3_ show the interactive effects of two factors, while the other can be navigated within the design space (Figure 3).

*Y*_1_ = 249.79 + 3.60*X*_1_ + 48.68*X*_2_ − 14.38*X*_3_ − 13.65*X*_1_*X*_3_ − 18.62*X*_2_*X*_3_ − 19.32*X*_2_^2^ + 57.99*X*_3_^2^ (8)

*Y*_2_ = 28.96 − 1.56*X*_1_ − 1.62*X*_2_ − 6.32*X*_3_ + 0.9025*X*_1_*X*_3_ − 4.05*X*_2_*X*_3_ (9)

*Y*_3_ = 1.62 − 0.1762*X*_1_ + 0.8587*X*_2_ − 1.46*X*_3_ − 0.2650*X*_1_*X*_2_ + 0.2375*X*_1_*X*_3_ − 0.7325*X*_2_*X*_3_ + 0.7300*X*_3_^2^ (10)

The positive regression coefficients in the RSM models indicate a direct relationship between a factor and a response. In contrast, negative values indicate an inverse relationship between a factor and the observed response. For example, In Equation (7), the size of the TO-FA-CS-Alg-NCs increased proportionally to the increase of *X*_1_ and *X*_2_; especially, *X*_2_ was the most significant effect (Figure 3a,c). These observed effects were due to an increase in the viscosity of the dispersion and the surface coating layers of the polymer as its concentration was increased in the dispersion [41]. In addition, Manaspon, Viravaidya-Pasuwat, & Pimpha [42] observed that the conjugation of FA with CS resulted in slightly larger NCs compared to the CS-coated NCs (without FA). On the other hand, increasing the concentration of *X*_2_ would remarkably increase the size of the NCs. The size of the TO-FA-CS-Alg-NCs increases by 48.7 nm with every 1% increase in *X*_2_. This effect could be due to the amphiphilic nature of poloxamer and the consequent increase in the size of the micelle core of the NCs, with additional quantities of TO being added into the formulation. Figure 3a,b show that an increase of *X*_3_ from a low to intermediate concentration decreased the size of the NCs. A lower surfactant concentration was insufficient to encapsulate the hydrophobic TO. Increasing *X*_3_ could have caused the formation of a stronger barrier of the micelle core by achieving the minimum size near the minimum level of *X*_3_ (within the experimental range). However, the further increase of *X*_3_ resulted in a remarkable increase in the size, probably due to the increased viscosity and near saturation of the aqueous phase with the micelle cores, consequently resulting in aggregation [43]. The excess amount of poloxamer in the dispersion could have interacted with the outer grafted-CS layer resulting in a bigger NC size.

The EE of TO-FA-CS-Alg-NCs was most significantly affected by *X*_3_ (poloxamer concentration) and had a minor effect on *X*_1_ and *X*_2_ as shown in Equation (8) and Figure 3d–f. The reduced capacity of the NCs to encapsulate TO was mainly because of *X*_3_. The miscibility of TO with the aqueous phases increased with the addition of poloxamer. However, an increasing *X*_3_ resulted in the saturation of the aqueous dispersion with micelles at the optimal concentration of *X*_3_ that was higher than the critical micelle concentration (CMC) of poloxamer [44]. The CMC of poloxamer is 2.8 × 10^−6^ M or approximately 3.5 × 10^−2^ mg/mL [45]. The concentration of 0.65% *w*/*v* that was used in the formulation was above the CMC of poloxamer 407. At this point, further increasing *X*_3_ would have a negligible impact on the encapsulation of TO. In another study, further increasing the concentration of poloxamer resulted in an insignificant increase in EE [42]. Due to the dispersion viscosity, these effects can be attributed to the hindered encapsulation of TO within the hydrophobic micelle core. The effects of *X*_1_ and *X*_2_ were then considered minor, where the grafted-CS polymer decreased the permeability and consequently minimized the leakage of TO from the core of the NCs by electrostatically interacting with the Alg matrix [37].

The LC of TO-FA-CS-Alg-NCs was directly affected by *X*_2_, indicating that LC increased with *X*_2_ (Equation (9); Figure 3h,i). The negative regression coefficients of *X*_1_ and *X*_3_ show their inverse effects on LC, with the most significant impact on LC demonstrated by *X*_3_ (Figure 3g,h). The increasing levels of *X*_1_ and *X*_3_ resulted in a lower LC because of the additional mass of the NCs conferred upon by the grafted polymer and surfactant, respectively. The latter figures also show the curvilinear effects of *X*_3_, with the maximum LC being achieved within minimum levels of *X*_3_ based on the design space. The LC was observed to increase with an increase in *X*_2_ due to the higher capacity of the surfactant-polymer system to accommodate additional amounts of TO but only until a certain point, as shown in Figure 3g.

### 3.4. Validation of Response Surface Model

To validate the generated RSM models, the TO-FA-CS-Alg-NCs were fabricated based on the optimized conditions of the FA-CS:Alg mass ratio (0.03:1), TO concentration (1.0% *w*/*v*), and poloxamer concentration (0.65% *w*/*v*) at desirability (D) of 0.961, which was closer to 1.0. The values of the observed responses were compared with the predicted responses for particle size, EE, and LC. A good agreement between the observed and predicted responses was found with all % error values below 10%, indicating the high predictive ability of the developed RSM models (Table 3).

Of particular interest is the optimal concentration for poloxamer, which was found to be 0.65%. This concentration was at the lower end of the 0.5 to 3.0% working range in the optimization experiments. Considering the biological applications of the optimized NCs, an increase in the concentration of poloxamer would increase its toxicity in normal cells both in vitro and in vivo [46]. Therefore, a minimum poloxamer concentration (0.65%) provided acceptable outcomes for this study.

### 3.5. Characterization of TO-FA-CS-Alg-NCs

**Figure 4:**
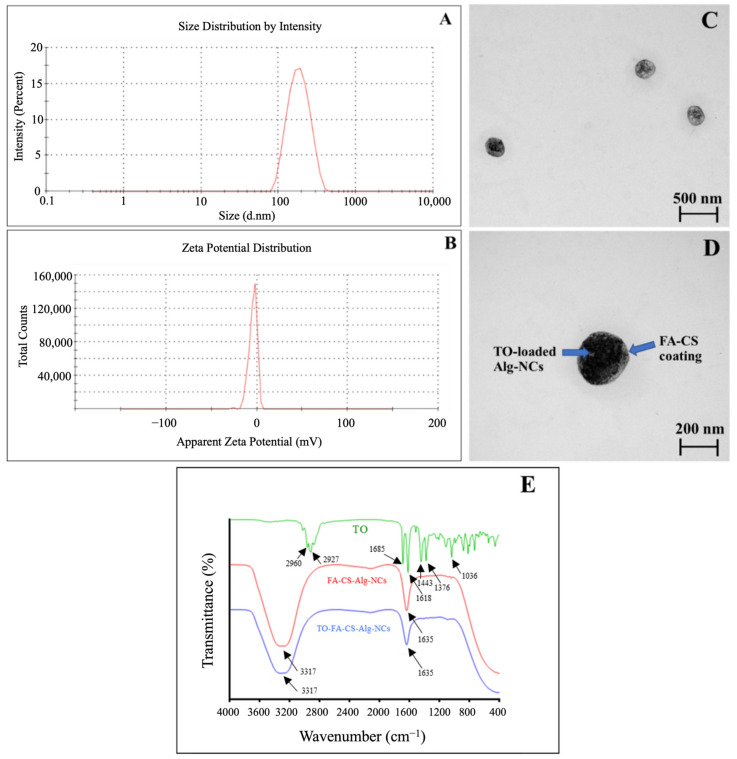
Physico-chemical characteristics of the optimized TO-FA-CS-Alg-NCs: (**A**) Size distribution by intensity; (**B**) Zeta potential distribution; (**C**,**D**) TEM images at 50,000 and 100,000× magnification, respectively; (**E**): FT-IR spectra of TO, FA-CS-Alg-NCs and TO-FA-CS-Alg-NCs.

The observed size of the optimized TO-FA-CS-Alg-NCs was 189 ± 4 nm (Table 3 and Figure 4A). The combined effects of the particle size and FA grafting of the outer CS chain facilitated a more accessible diffusion of the NCs into the tumor cells and significantly increased cellular internalization. This approximate size could also mean that the formulation can be administered intravascularly without concerns of embolization [47]. In addition, PDI was 0.192 ± 0.1, which is close to 0, indicating the highly homogenous distribution among the particles. This finding could also confirm by the TEM images of TO-FA-CS-Alg-NCs (Figure 4C,D). The NCs were spherical and had smooth surfaces with a particle size of approximately 200 nm. 

Zeta potential was also determined to predict the stability of NCs in the aqueous system based on their surface charge. In our previous study, the zeta potential of TO-CS-Alg-NCs (without FA conjugation) was −21.8 ± 1.1 [16]. Meanwhile, TO-FA-CS-Alg-NCs render a negative zeta potential value of −12.61 ± 2.3 (Figure 4B). Li et al. [27] also reported that the zeta potential was significantly decreased after the modification of CS with FA. This might be a partial substitution of -NH_2_ of CS with FA. Song et al. [48] justified the reduced surface charge was because of the reduction of the amount of protonated amino group of CS NPs, leading to a drop in its surface charge. The ligand molecules bound to the surface of NPs provide steric stabilization as an effect of repulsive forces (electrostatic interactions, steric exclusion, or hydration layer [49] and low PDI suggests an improvement in the steric hindrance of NPs, leads to steric stabilization consequently preventing the aggregation of NPs [50]. 

The FT-IR spectra of TO, TO-FA-CS-Alg-NCs, and FA-CS-Alg-NCs, were demonstrated in (Figure 4E). In the TO spectrum, the bands at 2960 cm^−1^ and 2927 cm^−1^ correspond to the -CH_3_ and -CH_2_ stretching vibration respectively. The peak at 1685 cm^−1^ and 1618 cm^−1^ contributed to the C=O vibration and aromatic ring stretching (C=C). Moreover, the –CH_2_ bending at 1443 cm^−1^, –CH_3_ bending indicated the presence of curcuminoids and C-OH stretching at 1034 cm^−1^ [51]. The peaks at 3317 cm^−1^ indicated the O-H and N-H stretching vibration in FA-CS-Alg-NCs and TO-FA-CS-Alg-NCs. The peak at 1635 cm^−1^ was due to the stretching vibration of the C=O bond in the amino group, and the peak at 1086 cm^−1^ was assigned to the C=N stretching [52]. The distinct peaks in FA-CS-Alg-NCs were also prominent in the TO-FA-CS-Alg-NCs, indicating the strong interaction between FA-CS and Alg in TO-loaded NC.

The observed results for EE and LC were expected as TO is a hydrophobic compound, and this study utilized hydrophilic polymers. Some studies using a polyelectrolyte complex of CS and Alg showed similar results for encapsulating hydrophobic compounds [38]. The loading capacity of TO in TO-CS-Alg-NCs was decreased from 3.4% [16] to approximately 2% (Table 3) when the FA was grafted to the CS backbone. This observation might be explained by the fact that several amino groups of CS were replaced by FA, the positive charges on the CS molecules that could attract the negative charges of Alg. Moreover, the spatial hindrance of folate derivatives on the CS backbone might inhibit the interaction between CS and Alg. When Alg was incorporated with FA-CS, it bound with the cationic residues of CS, which impairs electrostatic interactions between the polyelectrolytes with a consequent less compact structure, which was a faster displacement of the media molecules through the inner structure [53,54]. Di Martino, Trusova, Postnikov & Sedlarik (2018) and Yang, Lin et al. [53,54] also confirmed that the LC of the drug was strongly dependent on FA conjugation and Alg incorporation.

### 3.6. In Vitro Release Kinetics Study

A sustained-release profile, minimal release in the blood circulation, and maximal release in the tumor tissues are the necessary attributes of the nanoformulation [55,56]. In our previous study, we investigated the release of free TO and TO-loaded-ungrafted CS-Alg-NCs in both pH 7.4 and 5.5. The result showed a spontaneous release of free TO at the first 2 h, and almost 60% of the initially added TO was released after 7 h in both media [16]. Meanwhile, the release of TO from TO-CS-Alg-NCs (ungrafted) was slower than the unencapsulated TO in both media, and almost 50% of TO was released after 12 h and exhibited a sustained release. Furthermore, the TO release in low pH media (pH 5.5) was higher than in alkaline media (pH 7.4). This phenomenon can be explained by the increased solubilization of CS in acidic media, causing the leakage of the encapsulated TO through the porous CS-Alg-NCs into the releasing media. On the other hand, TO was slowly released from the CS-Alg-NCs in alkaline media due to CS shrinkage, preventing encapsulated TO from CS-Alg-NCs [41].

**Figure 5:**
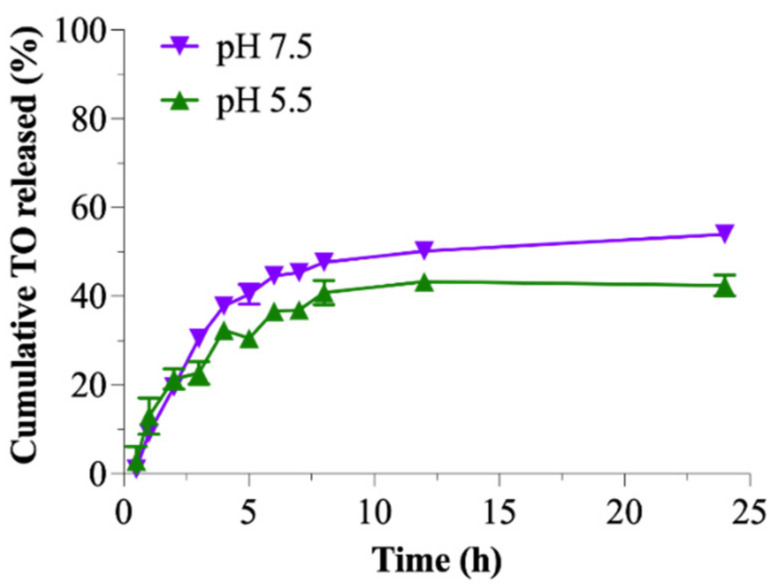
Cumulative release of TO from the TO-FA-CS-Alg-NCs in pH 5.5 and 7.4.

However, a significant difference in the release rate of TO from TO-FA-CS-Alg-NCs in two different pH conditions was observed after 3 h, wherein higher TO was released at pH 7.5 (Figure 5). When comparing the ungrafted CS-Alg and FA-CS-NCs, it clearly showed that the pH of the dissolution medium was found to have greatly affected the release rate of TO from the NCs. From our previous report, a higher % TO was released from ungrafted CS-Alg-NCs in pH 5.5 than in 7.4 owing to the protonation of NH_2_ groups and the high solubility of CS in acidic sink conditions [16]. In contrast, the release rate of TO-FA-CS-Alg-NCs increased with the increase in pH of the dissolution medium. This was possible due to the different chemical structures on the backbone of CS thereby altering its physicochemical properties, particularly the solubility of the polymer in an acidic medium. The same findings of higher drug release from FA-CS NPs in basic conditions were also reported by Lazer et al. [57]. Nonetheless, these results demonstrate that FA-CS-Alg-NCs can serve as sustained and controlled release carriers for oral and intravenous administration of TO [58].

The DDSolver was used to evaluate the release profile of TO from the TO-FA-CS-Alg-NCs and determine the model that best describes this release profile. In two media, the in vitro release data of TO from TO-FA-CS-Alg-NCs was fitted to Korsmeyer-Peppas. This model showed the highest coefficient of determination (R^2^), lowest AIC, and maximal MSC values (Table S3). Additionally, the Korsmeyer-Peppas model showed that the release exponent (n) of TO from the TO-FA-CS-Alg-NCs in the two media was in the range of 0.345–0.387 (Table S3). These data indicate that the drug release followed the Fickian diffusion-controlled release pattern (n < 0.5), which could be inferred as the controlling factor for the diffusion of TO through the pore of FA-CS-Alg-NCs based on the TO concentration gradient, that can be used for a controlled and sustained drug release dosage form. Accordingly, these results can be used to further design and develop TO-FA-CS-Alg-NCs for the required administration route [52].

### 3.7. Protein Binding

**Figure 6:**
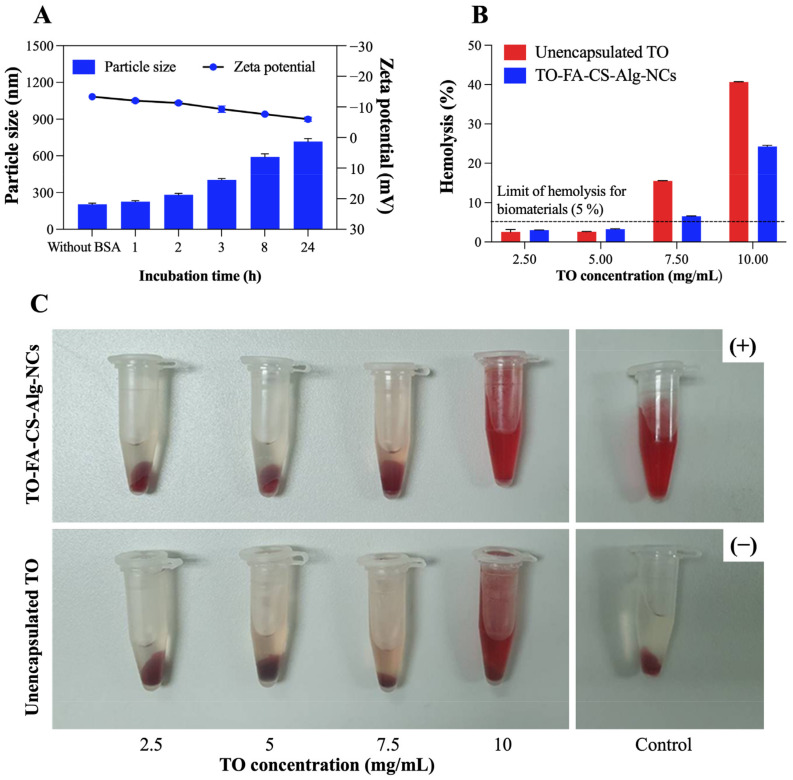
(**A**) The change in particle size and zeta potential of TO-FA-CS-Alg-NCs after 1–24 h incubation with BSA at 25 °C; In vitro hemolysis assay of TO-FA-CS-Alg-NCs and free TO; (**B**) hemolysis percentage of different TO concentrations; (**C**) Images of RBC treated with TO-FA-CS-Alg-NCs and free TO. Data are expressed as mean ± SD (n = 3).

The binding of BSA protein onto TO-FA-CS-Alg-NCs was investigated in this study, and the findings are shown in Figure 6A. After incubation with BSA for 1–2 h, a slight difference in particle size (281 ± 13 nm) and zeta potential (−10.9 ± 0.7 mV) compared to non-BSA incubation (particle size is 203 ± 11 nm and zeta potential is −13.7 ± 0.9 mV, respectively) was observed. The large difference in particle size and zeta potential was observed after 8 h of incubation, and the particle size and zeta potential at the end of incubation after 24 h were 717.2 ± 24 nm and −5.97 ± 1.7 mV, respectively. The ionic interaction and charge neutralization between BSA and nanoparticles may cause an increased particle size and a lower zeta potential [59], and our results were in agreement with the report described by Katas et al. [60]. Furthermore, no aggregation of nanoparticles was found after incubation with BSA, which may indicate that the nanoparticles created could be employed in preclinical and clinical applications.

### 3.8. In Vitro Hemolytic Compatibility

As seen in Figure 6B,C, the hemolytic activity was observed to be concentration-dependent for both sample groups, suggesting higher levels of hemoglobin were released from the rupture of red blood cells induced by the higher TO concentration in both samples. Except at TO concentrations of 7.5 mg/mL and 10 mg/mL, both samples were considered non-hemolytic activity since the hemolysis percentage was less than 5% according to ISO/Technical Report 7406 guidelines, and nanoparticle or material hemolytic percentages are limited to 5% [61]. The hemolysis percentage of TO-FA-CS-Alg-NCs (26%) was 1.6 times higher than free TO (41%) at the highest tested TO concentration (10 mg/mL), which may indicate that the safety of TO has been enhanced by the encapsulation in FA-CS-Alg-NCs. These findings could also be attributed to CS and Alg polymers’ low toxicity and hemocompatibility [62]. Our findings suggest that both free TO and TO-FA-CS-Alg-NCs exhibited non-hemolytic activities (5%), except at the highest TO concentration tested (7.5–10 mg/mL), implying that they are safe for human use and warrant further investigation for blood contact applications. 

### 3.9. Cellular Uptake

**Figure 7:**
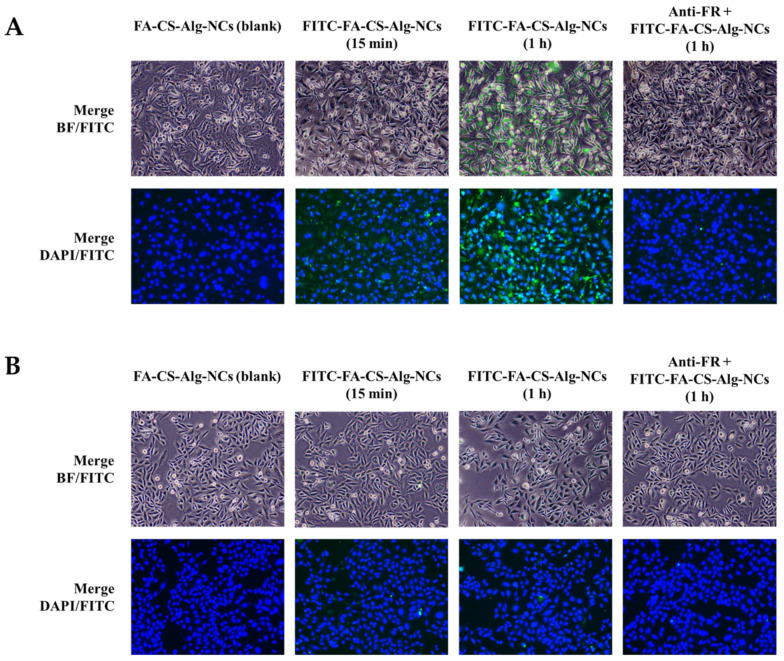
Cellular uptake of FITC-FA-CS-Alg-NCs in (**A**) MDA-MB-231 and (**B**) MCF-10A cells at 15 min and 1 h incubation times. Cells were also pre-treated with anti-human folate receptors αβ antibody (anti-FR) for 30 min prior to FITC-FA-CS-Alg-NCs treatment for 1 h. The green color indicates the uptake of FITC-FA-CS-Alg-NCs, while the blue color indicates the nuclei stained with DAPI. Merged images between brightfield (BF)-FITC and DAPI-FITC show the internalization of FITC-FA-CS-Alg-NCs in the cells.

The qualitative cellular uptake of FITC-FA-CS-Alg-NCs was evaluated on MDA-MB-231 and MCF-10A cells using a fluorescence microscope. The images were obtained by setting a constant laser intensity and exposure time to compare the cellular uptake of each of the samples. Brightfield (BF) images and FITC channel or DAPI staining with FITC channel were merged to evaluate the internalization of FITC in the cytoplasm of each cell. Blank FA-CS-Alg-NCs showed no green, fluorescent signal, while the increasing incubation time of FITC-FA-CS-Alg-NCs showed a brighter green, fluorescent intensity in MDA-MB-231 cells (Figure 7A). To further evaluate the affinity of FITC-FA-CS-Alg-NCs to FR-positive TNBC, the cells were pre-saturated with anti-human FRαβ antibody prior to the addition of treatments. Interestingly, blocking the FR diminished the cellular uptake of FITC-FA-CS-Alg-NCs in MDA-MB-231 and MCF-10A cells. Moreover, in low FR-expressing MCF-10A cells, the intracellular green, fluorescent signal of FITC was found to be remarkably reduced than in MDA-MB231 cells, showing that the uptake was mainly dependent on the expression level of FRs (Figure 7B). Hence, these results clearly suggest the enhanced tumor cell targeting effect and cellular uptake of FA-CS-Alg-NCs through FR-mediated endocytosis, which facilitated the entry of FITC into FR-positive TNBCs.

### 3.10. FR Expression Level of Cytotoxicity

The cancer cell lines, MDA-MB-231 and MCF-7 were chosen to demonstrate the active cellular targeting of FA-CS-Alg-NCs against FR-expressing human breast cancer cells. The MDA-MB-231 and MCF-7 cells are typically used in in vitro research because of their differences in origin, survival, and recurrence rates. The MCF-7 comes from nonmetastatic human breast adenocarcinoma and is positive for both estrogen (ER) and progesterone receptor (PR). While MDA-MB-231 cells belong to the TNBC subtype that lacks (ER−), (PR−), and human epidermal growth factor receptor (HER2−) [63]. Both MDA-MB-231 and MCF-7 cells express a high density of FRs, but MDA-MB-231 cells express FR-α to a greater extent than MCF-7 [63]. Earlier reports show that MDA-MB-231 cells have 1.76 times more FR-α than MCF-7 cells [64]. Hence, by using these cell lines, we would be able to differentiate its responses to targeted TO delivery to FRs.

**Figure 8:**
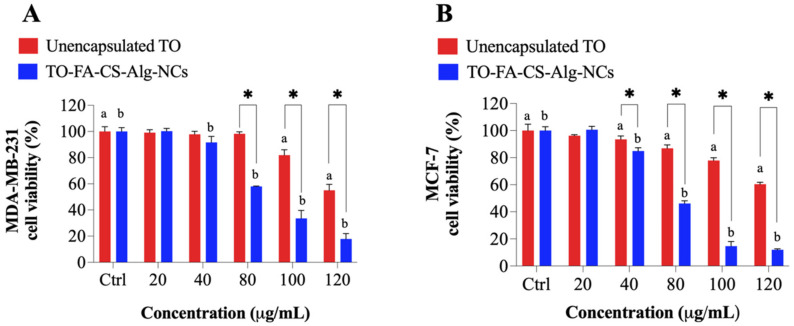
The viability of (**A**) MDA-MB-231 and (**B**) MCF-7 treated with unencapsulated TO and TO-FA-CS-Alg-NCs at different concentrations. ^a,b^ *p* < 0.05 compared to the control (^a^ DMSO in red and ^b^ empty FA-CS-ALG-NCs in blue), * *p* < 0.05 between unencapsulated TO and TO-FA-CS-ALG-NCs.

The result of the experiment showed that both breast cancer cells express FRs on their surfaces. The FR expression levels of MDA-MB-231 were 1.6-fold than that of MCF-7 (Figure S1). An MTT cytotoxicity assay was performed to determine the enhanced anticancer activity of TO-FA-CS-Alg-NCs and unencapsulated TO on MDA-MB-231 and MCF-7 breast cancer cells (Figure 8). Statistical analysis showed that the TO-FA-CS-Alg-NCs had more potent toxicity to MDA-MB-231 at 40 µg/mL, while the unencapsulated TO demonstrated significant toxicity at 100 µg/mL concentration (Figure 8A). Moreover, significant toxicity to MCF-7 was observed in both unencapsulated TO and TO-FA-CS-Alg-NCs at a concentration of 40 µg/mL (Figure 8B). 

The viabilities of MDA-MB-231 and MCF-7 with the treatments for each concentration were also compared. TO-FA-CS-Alg-NCs were more toxic toward MDA-MB-231 and MCF-7 at 80 and 40 µg/mL, respectively, compared to unencapsulated TO. The higher cytotoxicity of TO-FA-CS-Alg-NCs may be ascribed to the receptor-mediated endocytosis by both breast cancer cells, which increases the concentration of TO in the intracellular environment. To further prove this hypothesis, the IC_50_ values of the unencapsulated TO and TO-FA-CS-Alg-NCs are presented in Table 4. The results show the IC_50_ values of TO-FA-CS-Alg-NCs against both cell lines significantly differed from the unencapsulated TO. Remarkably, the IC_50_ values of TO-FA-CS-Alg-NCs were significantly lower than TO-CS-Alg-NCs (without FA) from our previous study [16] in both breast cell lines, which indicated the targeting advantage of FA-CS [65]. The MCF-7 cells seem to be more sensitive to the cell viability effect of TO than MDA-MB-231 in both TO-CS-Alg-NCs and TO-FA-CS-Alg-NCs.

Overall, TO-FA-CS-Alg-NCs exhibited a significantly higher toxicity level than TO-CS-Alg NCs and unencapsulated TO against both breast cancer cells, signifying that FA-CS-Alg-NCs could enhance the toxicity effects of TO against both invasive MDA-MB-231 and MCF-7 breast cancer cells. The higher cytotoxicity of TO-FA-CS-Alg-NCs could be attributed to the interaction of the FA-CS with the FR that is overexpressed on the membrane of MDA-MB-231 and MCF-7 [48].

### 3.11. Storage Stability Studies

**Figure 9:**
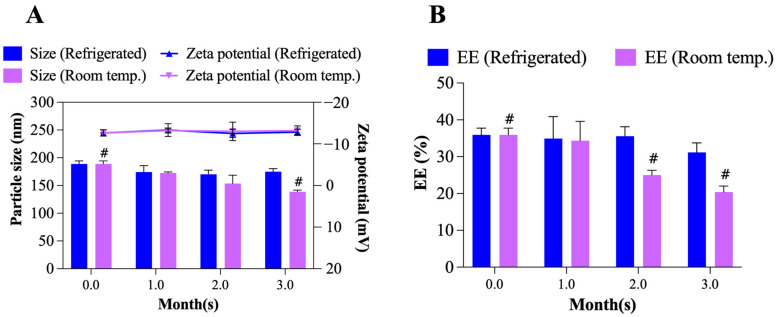
Storage stability of TO-FA-CS-Alg-NCs: (**A**) particle size and zeta potential, (**B**) EE after 3 months of storage in different temperature conditions. **^#^** *p*< 0.05 compared to Month 0.

The changes in particle size, zeta potential and EE of TO-FA-CS-Alg-NCs were evaluated for 3 months under different storage conditions (4 °C and room temperature) as shown in Figure 9A,B. At 4 °C, the particle size and zeta potential did not significantly change during storage, while its EE slightly changed from 35.9 ± 1.5% to 31.2 ± 2.1% after 3 months. Moreover, TO-FA-CS-Alg-NCs stored at room temperature showed a significant decrease in the particle size (189 ± 5 nm to 139 ± 1 nm) in the 3rd month. This can be due to the leakage of TO from the FA-CS-Alg-NCs that is evident in the significant reduction of % EE (35.9 ± 1.5% to 20.4 ± 2.7%) and the formation of cloudy solution starting in the 2nd month of storage at room temperature. The same findings were reported by Alam et al. [66], where the leakage of the drug from the NPs was due to accelerated hydrolysis of a polymer matrix that caused its erosion when stored in hotter conditions [66]. Hence, these results strongly suggest that TO-FA-CS-Alg-NCs had better stability under 4 °C storage conditions for 3 months.

## 4. Conclusions

The conjugation of FA with CS involved an amidation reaction using NHS/DCC chemistry with a degree of substitution of 12.86%. TO-FA-CS-Alg-NCs were prepared via ionotropic gelation by the electrostatic interaction between the -COOH groups of Alg and -NH_2_ of CS. The fabrication of TO-FA-CS-Alg-NCs was successfully optimized using the BBD rendering good particle size (189 nm) with modest aggregation and relatively high EE and LC. Cell viability assay revealed that the TO-FA-CS-Alg-NCs were more toxic than TO-CS-Alg NCs (without FA) and unencapsulated TO against MDA-MB-231 and MCF-7 breast cancer cell lines due to its FR targeting mechanism. In prospect, the TO-FA-CS-Alg-NCs can be further evaluated in a preclinical study for breast cancer therapy.

## Figures and Tables

**Table 1:** Factors and responses for the BBD.

Factors	Levels		
	Low	Medium	High
*X*_1_ = FA-CS:Alg mass ratio	0.03:1	0.05:1	0.07:1
*X*_2_ = TO concentration (% *w*/*v*)	1.0	1.5	2.0
*X*_3_ = Poloxamer concentration (% *w*/*v*)	0.5	1.75	3.0
**Responses**	**Constraints**		
*Y*_1_ = Particle size (nm)	Minimum		
*Y*_2_ = EE (%)	Maximum		
*Y*_3_ = LC (%)	Maximum		

**Table 2:** Box-Behnken experimental design matrix and response values for the optimization of TO-FA-CS-Alg-NC formulation.

	Factors			Responses		
Run	*X*_1_	*X*_2_	*X*_3_	*Y*_1_	*Y*_2_	*Y*_3_
1	0.03	1.0	1.75	175 ± 21	33.1 ± 1.2	0.88 ± 0.01
2	0.07	1.0	1.75	188 ± 8	28.6 ± 1.0	1.04 ± 0.05
3	0.03	2.0	1.75	279 ± 12	29.2 ± 3.0	2.96 ± 0.36
4	0.07	2.0	1.75	279 ± 16	25.6 ± 1.7	2.06 ± 0.31
5	0.03	1.5	0.50	310 ± 12	37.1 ± 4.2	4.17 ± 0.50
6	0.07	1.5	0.50	345 ± 8	33.3 ± 3.4	3.36 ± 0.37
7	0.03	1.5	3.00	297 ± 9	23.3 ± 0.8	0.81 ± 0.14
8	0.07	1.5	3.00	278 ± 9	23.0 ± 1.1	0.95 ± 0.18
9	0.05	1.0	0.50	230 ± 11	33.2 ± 2.2	2.18 ± 0.08
10	0.05	2.0	0.50	365 ± 19	38.3 ± 7.0	5.53 ± 0.20
11	0.05	1.0	3.00	250 ± 14	28.0 ± 0.7	0.69 ± 0.12
12	0.05	2.0	3.00	310 ± 18	16.9 ± 1.4	1.11 ± 0.51
13	0.05	1.5	1.75	244 ± 19	28.2 ± 1.8	1.53 ± 0.39
14	0.05	1.5	1.75	248 ± 16	28.8 ± 1.4	1.4 ± 0.32
15	0.05	1.5	1.75	258 ± 19	28.0 ± 1.4	1.47 ± 0.11

*X*_1_: FA-CS:Alg mass ratio; *X*_2_: TO (% *w*/*v*); *X*_3_: poloxamer (% *w*/*v*); *Y*_1_: particle size (nm); *Y*_2_: EE (%); *Y*_3_: LC (%).

**Table 3:** Validation of the prediction capability of the RSM models.

Optimized Condition	Response	Predicted Response	Observed Response *	%Error
*X*_1_: 0.03:1	Y_1_ (nm)	207	189 ± 4	8.6
*X*_2_*:* 1.0%	Y_2_ (%)	34.9	35.9 ± 13.5	2.7
*X*_3_: 0.65%	Y_3_ (%)	2.08	1.82 ± 0.39	0.12

*X*_1_: FA-CS:Alg mass ratio; *X*_2_: TO concentration (% *w*/*v*); *X*_3_: Poloxamer concentration (% *w*/*v*); * Values are expressed as mean ± SD, n = 3.

**Table 4:** Mean IC_50_ values of the unencapsulated TO and formulated TO.

	IC_50_ (µg/mL).	
Treatments	MDA-MB-231	MCF-7
Unencapsulated TO	122.8 ± 2.8 ^c,d^	139.7 ± 4.0 ^f,g^
TO-FA-CS-Alg NCs	85.3 ± 2.8 ^c,e^	71.1 ± 1.1 ^f,h^
TO-CS-Alg-NCs [16]	99.1 ± 3.4 ^d,e^	82.9 ± 4.4 ^g,h^

The same letter within each cell line signifies statistically significant at *p* < 0.05.

## Data Availability

All the data are available within the manuscript.

## Supplementary Materials

The following supporting information can be downloaded at: https://www.mdpi.com/article/10.3390/pharmaceutics15010110/s1, Table S1: Assignment of 1H-NMR spectral data of FA-CS conjugate in d6-DMSt; Table S2: Summary of the regression analyses of the responses; Table S3: Release kinetics of TO from TO-FA-CS-Alg-NCs; Figure S1: Folate expression levels in MDA-MB-231 and MCF-7 cell lines.
