# Supplementary material for: Folic Acid-Grafted Chitosan-Alginate Nanocapsules as Effective Targeted Nanocarriers for Delivery of Turmeric Oil for Breast Cancer Therapy
Source: Pharmaceutics. 2022 Dec 28;15(1):110. doi: 10.3390/pharmaceutics15010110 (PMC9862360; doi:10.3390/pharmaceutics15010110)
Supplement: Supplementary file 1 [file pharmaceutics-15-00110-s001.zip › pharmaceutics-2088162-supplementary.pdf]

pharmaceutics

MDPI

Folic acid-grafted chitosan-alginate nanocapsules as effective targeted nanocarriers for delivery of turmeric oil for breast cancer therapy

Htet Htet Moe San, Khent Primo Alcantara, Bryan Paul I. Bulatao, Feuangthit Niyamissara Sorasitthiyanukarn, Nonthaneth Nalinratana, Apichart Suksamrarn, Opa Vajragupta, Pranee Rojsitthisak, Pornchai Rojsitthisak

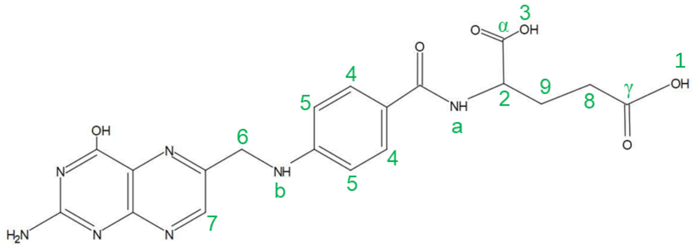

FA

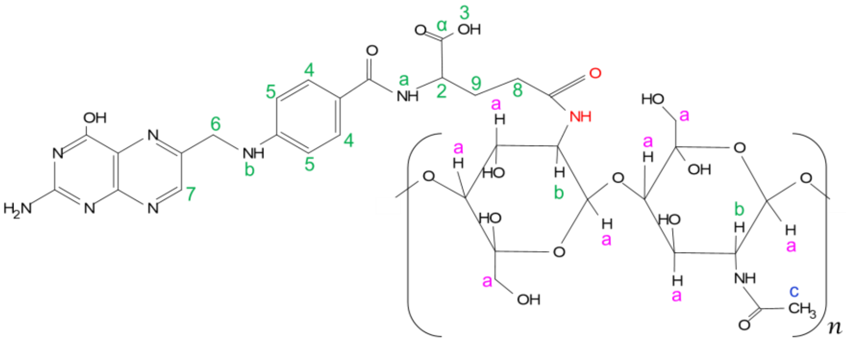

FA-CS

Table S1. Assignment of ^{1}H-NMR spectral data of FA and FA-CS conjugate in DMSO-d_6

Position	^{1}H NMR Shift (ppm)	
	FA	FA-CS
NH (a)	8.13	-
NH (b), NH_2	6.93	6.90
1 -COOH	11.40	-
2	4.30	4.50
3 -COOH	12.30	10.60
4	7.66	7.63
5	6.60	6.66
6	4.50	4.46
7	8.60	8.65
8	2.30	2.30
9	2.14	2.14

Table S2. Summary of the regression analyses of the responses.

Response	Suggested model	*p*-value, overall model	*p*-value, lack-of-fit	R^2	Adjusted R^2	Predicted R^2	Adequate precision
Y_1	Quadratic	< 0.0001	0.5347	0.9894	0.9787	0.9220	34.6723
Y_2	2FI	< 0.0001	0.2620	0.9885	0.9820	0.9635	44.0165
Y_3	Quadratic	< 0.0001	0.1039	0.9930	0.9859	0.9641	38.5856

Table S3. Release kinetics of TO from TO-FA-CS-Alg-NCs

Model	Media	Parameter	R^2 adjusted	AIC	MSC
Zero order ($F = k_0 \cdot t$)	pH 5.5	$k_0 = 2.976$	-0.7961	90.1318	-0.9216
	pH 7.4	$k_0 = 3.633$	-0.3075	93.3060	-0.4488
First order ($F = 100 \cdot e^{-k_1 t}$)	pH 5.5	$k_1 = 0.053$	0.0774	82.8555	-0.2601
	pH 7.4	$k_1 = 0.075$	0.5321	81.9675	0.5820
Higuchi ($F = k_H \cdot t^{0.5}$)	pH 5.5	$k_H = 12.116$	0.6697	71.6241	0.7609
	pH 7.4	$k_H = 14.603$	0.7513	75.0397	1.2118
[a]Korsmeyer-Peppas ($F = k_{KP} \cdot t^n$)	pH 5.5	$k_{KP} = 17.166$, $n = 0.345$	0.7879	67.3280	1.1515
	pH 7.4	$k_{KP} = 18.819$, $n = 0.387$	0.7863	74.2195	1.2863
Hixson-Crowell ($F = 100 \cdot [1 - (1 - k_{HC} \cdot t)^3]$)	pH 5.5	$k_{HC} = 0.015$	-0.1687	85.4417	-0.4952
	pH 7.4	$k_{HC} = 0.021$	0.34170	85.7354	0.2394

[a]Best-fit release kinetics model for TO-FA-CS-Alg-NCs. F is the fraction (%) of drug released at time t; k_0 is the zero-order release constant; k_1 is the first-order release constant; k_H is the Higuchi release constant; k_{KP} is the release constant incorporating structural and geometric characteristics of the drug-dosage form; n is the diffusional exponent indicating the drug-release mechanism; k_{HC} is the Hixson-Crowell release constant.

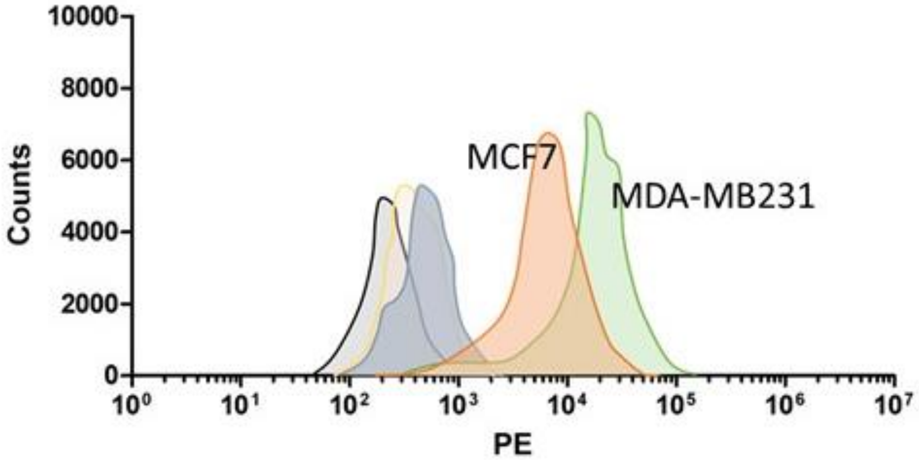

Figure. S1 Folate expression levels in MDA-MB-231 and MCF-7 cell lines
